# Determining rotational dynamics of the guanidino group of arginine side chains in proteins by carbon-detected NMR†
†Electronic supplementary information (ESI) available. See DOI: 10.1039/c7cc04821a


**DOI:** 10.1039/c7cc04821a

**Published:** 2017-08-25

**Authors:** Karola Gerecht, Angelo Miguel Figueiredo, D. Flemming Hansen

**Affiliations:** a Institute of Structural and Molecular Biology , Division of Biosciences , University College London , London , WC1E 6BT , UK . Email: d.hansen@ucl.ac.uk

## Abstract

Arginine residues are imperative for many active sites and protein-interaction interfaces. A new NMR-based method is presented to determine the rotational dynamics around the N_ε_–C_ζ_ bond of arginine side chains. An application to a 19 kDa protein shows that the strengths of interactions involving arginine side chains can be characterised.

## Abstract 2

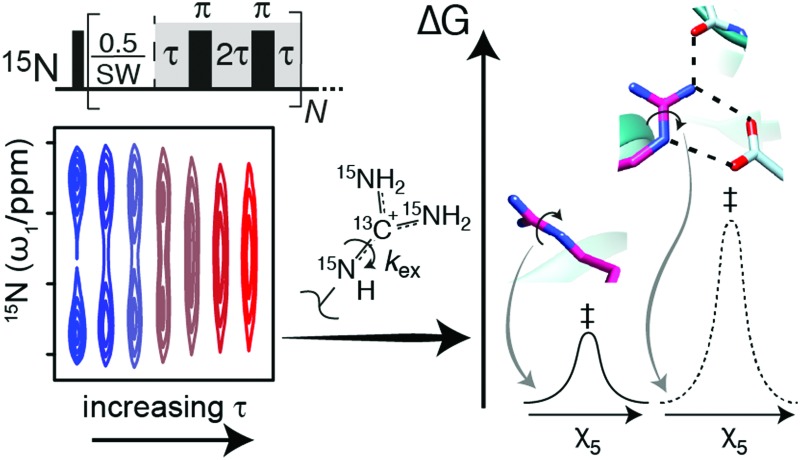
A new NMR-based method is presented to determine the rotational dynamics around the N_ε_–C_ζ_ bond of arginine to characterise the interactions mediated by arginine side chains.

## 

Internal motions of proteins and their side chains allow a perpetual sampling of various conformations that have been shown to be important for both protein–ligand interactions and enzyme catalysis.^1^–^3^ Internal motions occur on a broad range of timescales, ranging from picoseconds to seconds, and nuclear magnetic resonance (NMR) spectroscopy has proven a powerful experimental technique to quantify such dynamics with atomic resolution.^4^

Arginine side chains and their motions are particularly important for protein function because of the high p*K*_a_^5^ of the terminal guanidino group and its five possible hydrogen bond formation sites (Fig. S1, ESI†). These features drive the formation of bidentate salt bridges,^6^ cation–π interactions^7^ and hydrogen bonding networks.^8^ Hence, arginine residues feature in DNA- and RNA-binding proteins,^9^–^11^ phosphate binding modules such as the SH2 domain,^9^ receptors such as glutamate receptors,^10^ and in many enzymatic active sites.^12^ Arginine residues that form interactions *via* their guanidino group generally experience a restriction of the rotational motion around the N_ε_–C_ζ_ bond; with the exception of end-on interactions with glutamate or aspartate.^6^ Therefore, a method to quantify the N_ε_–C_ζ_ rotational dynamics of arginine side chains will allow insights into the diverse range of processes that arginine side chains are involved in and in many cases allow for a quantification of the interactions formed. Below we present an NMR method, based on ^13^C-detection, to quantify the N_ε_–C_ζ_ rotational dynamics and thus to allow a quantification of interactions formed by the arginine guanidino group under physiological conditions.

Carbon-13 detection inherently suffers from a lower signal intensity in NMR spectra due to the fourfold lower gyromagnetic ratio of ^13^C compared to ^1^H. However, ^13^C-detection becomes advantageous, when the NMR signals of the protons at the site of interest are severely broadened, for example by rapid exchange with the bulk solvent.^12^–^14^ At ambient temperature and at neutral pH, the H_η_ amine protons of the arginine side chain exchange rapidly with the solvent,^15^ which renders conventional ^1^H–^15^N proton-detected NMR experiments ill-suited to detect them. To circumvent this problem, ^13^C-detection is used here to probe the ^15^N_η_. The NMR pulse scheme developed is shown in Fig. 1 and it is based on a recently developed method for characterising the ^13^C_ζ_–^15^N_ε_ spin pairs.^16^ Briefly, magnetisation is initially transferred from ^13^C_ζ_ to the two ^15^N_η_*via* the one-bond ^13^C_ζ_–^15^N_η_ scalar couplings. The considerable difference in the chemical shift ranges of ^15^N_η_ (∼71 ppm) and ^15^N_ε_ (∼85 ppm)^17^ allows a selective transfer to the two terminal ^15^N_η_ without a transfer to ^15^N_ε_ using a selective pulse. Subsequent ^15^N chemical shift evolution followed by a second INEPT allows for ^13^C_ζ_–^15^N_η_ correlation spectra.

**Fig. 1:**
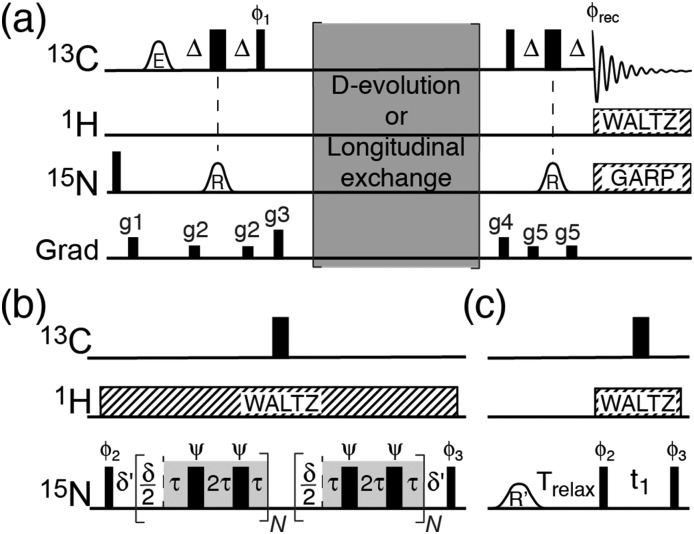
(a) Pulse schemes for characterising the N_ε_–C_ζ_ rotational dynamics of arginine side chains in proteins. The carrier positions are: ^1^H 7 ppm, ^13^C: 156 ppm, ^15^N 71 ppm. Narrow bars represent 90° pulses and wide bars represent 180° pulses. The delay *Δ* is 1/(8*J*_C_ζ_–N_η__) = 6.3 ms, and *δ* = 1/SW, where SW is the sweep width in the ^15^N dimension. Shaped pulses are represented by bell-shapes with letters specifying the shape (R: RE-BURP, E: E-BURP^29^). Phases are *x* unless stated otherwise. The phase cycle used is *φ*1: 4(*y*), 4(–*y*); *φ*2: *x*, –*x*; *φ*3: 2(*x*), 2(–*x*); *φ*_rec_: *x*, 2(–*x*), *x*, –*x*, 2(*x*), –*x*. Decoupling sequences are represented by striped boxes indicating the type of decoupling: WALTZ16 (4 kHz), GARP4 (0.7 kHz). Gradients of 1 ms are applied with strength of g1: 9.5 G cm^–1^, g2: 3.9 G cm^–1^, g3: 26.2 G cm^–1^, g4: 18.4 G cm^–1^, g5: 7.2 G cm^–1^. The first ^15^N pulse and gradient g1 are applied to purge longitudinal ^15^N magnetisation. (b) D-Evolution element. The phase *ψ* follows an XY16^30^ phase cycle and is incremented for each 180° pulse and the *τ* delay is in the range from 70 μs to 1 ms (see text). *δ*′ is to compensate for chemical shift evolution during the 90° ^15^N pulses and the 180° ^13^C pulse, *N* = *t*_1_/*δ*. D-Evolution experiments performed with a high-power ^1^H CW^31^ decoupling or with WALTZ decoupling lead to identical exchange rates. We therefore prefer to perform the D-evolution experiments with a WALTZ decoupling, which can be applied at a lower power to reduce heating effects. (c) Longitudinal exchange element. The shaped pulse R′ is 10 ms and made selective for only one of the two ^15^N_η_ resonance (see text).

The 19 kDa protein T4 lysozyme with the cavity forming mutation L99A^18^,^19^ (T4L99A) serves as a good and realistic model protein for arginine studies because it is well-characterised and contains 13 arginine residues. A two dimensional ^13^C_ζ_–^15^N_η_ correlation spectrum obtained using the pulse scheme in Fig. 1 is shown in Fig. 2, where cross peaks were assigned based on the ^13^C_ζ_ chemical shifts (Fig. S2, ESI†). Five of the 13 arginine ^13^C_ζ_ are well resolved and an obvious difference in the dynamics of the ^15^N_η_ groups is immediately apparent. For example, two separate peaks are observed for the two ^15^N_η_ of R95 and R145, showing a slower exchange for these residues. By contrast, the two ^15^N_η_ resonances of R14 only give rise to one broad peak, thereby indicating that the two amine groups are exchanging faster. Thus, the ^13^C_ζ_–^15^N_η_ spectrum is sensitive to the exchange between the two ^15^N_η_, that is, the rotational dynamics around the N_ε_–C_ζ_ bond. As detailed below a quantification of this exchange reports on the strength of interactions formed by the arginine side chain.

**Fig. 2:**
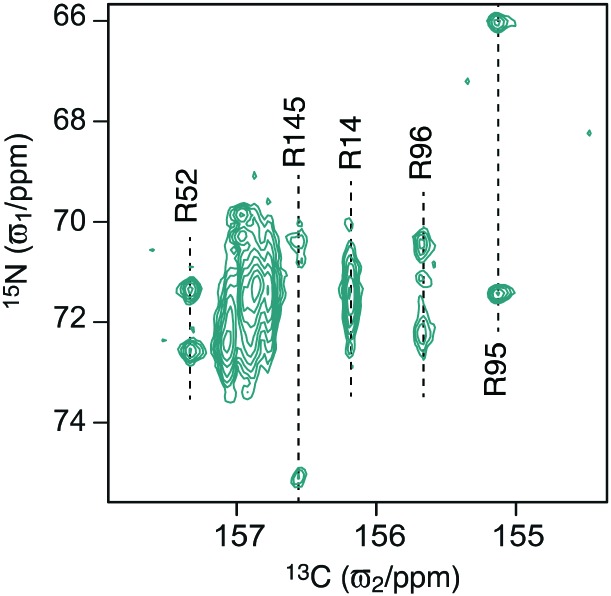
Carbon-detected ^13^C_ζ_–^15^N_η_ spectrum of T4L99A. The spectrum was recorded without D-evolution (without the grey box in Fig. 1b) at 18.8 T and at a temperature of 298 K. The chemical shift assignment was obtained *via* the ^13^C_ζ_ chemical shifts and the previous assignment of the ^13^C_ζ_–^15^N_ε_ correlation spectrum.^16^

Various techniques have been developed to study chemical exchange processes on different time scales using NMR spectroscopy. Carr–Purcell–Meiboom–Gill (CPMG)^20^,^21^ relaxation dispersion spectroscopy is applicable for exchange processes in the ms–μs regime^22^ whereas longitudinal exchange spectroscopy (EXSY) is widely applied to investigate processes with rates in the seconds range. We decided here to implement two variants of these experiments to determine the rotational dynamics of arginine guanidino groups, that is, (i) divided-evolution (D-evolution)^23^ within the ^15^N chemical shift evolution period of the ^13^C_ζ_–^15^N_η_ experiment (Fig. 1b) and (ii) a longitudinal exchange experiment based on selective pulses^24^ (Fig. 1c). D-Evolution has previously been used to improve the signal intensity of exchange-broadened resonances^23^ as well as in combination with CPMG relaxation dispersion experiments to obtain accurate exchange rates and populations of low-populated states.^25^ Briefly, each time increment in the chemical shift evolution period is separated by a CPMG element. This results in an apparent shift of the exchange process towards the fast-exchange regime and consequently a different peak shape.

Free [^15^N_4_,^13^C_6_]-arginine dissolved in 50%/50% MeOH/H_2_O under acid conditions was used to evaluate under which conditions reliable exchange rates for the rotational dynamics of arginine guanidino groups, *k*_ex_, can be obtained using D-evolution. D-evolution experiments were recorded over a range of temperatures and using six different *τ* delays in the range from 70 μs to 1 ms. The D-evolution spectra obtained at 273 K are shown in Fig. 3. At 273 K the exchange between the two ^15^N_η_ for free arginine is near the slow exchange regime, such that two separate peaks for the two ^15^N_η_ nuclei are observed both without D-evolution and for small *τ*. For larger *τ* delays these two peaks coalesce into the intermediate exchange regime and further into a single peak for larger *τ* delays. It should be noted that the extent of apparent relaxation and line broadening increases when *τ* is increased, which counteracts the peak sharpening effect; Fig. 3a and b. However, our motivation here is to use D-evolution to determine the exchange rate constant and not that of improving signal-to-noise.

**Fig. 3:**
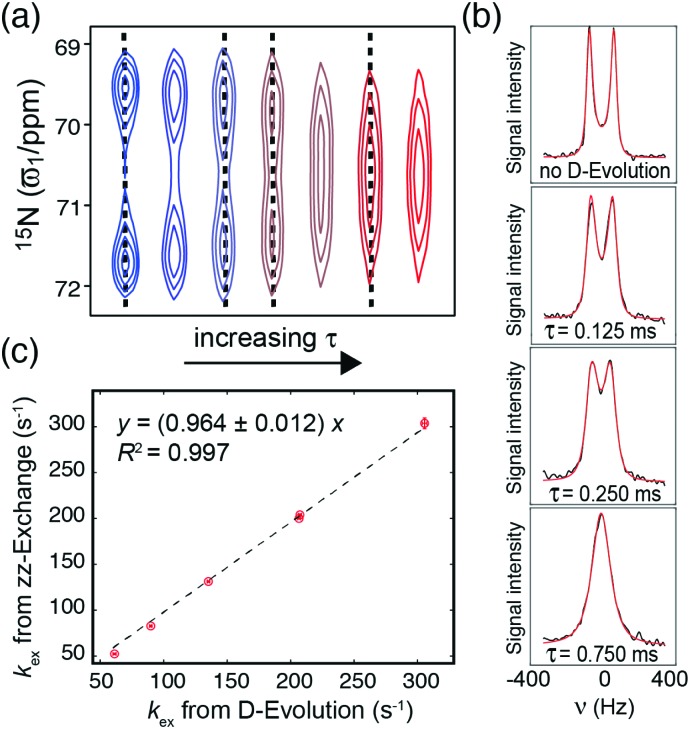
(a) ^13^C_ζ_–^15^N_η_ D-evolution spectra of free arginine showing that the peaks are moving closer and coalesce upon increasing *τ*. Buffer contained 50% MeOH, 50% H_2_O and spectra were recorded at 273 K and at 14.1 T. (b) Least-squares analysis of the spectra in (a) (dotted lines); with experimental spectra in black and theoretical back-calculated spectra in red. The exchange rate constant *k*_ex_ was extracted from the experimental spectra with different *τ* using theoretically derived spectra and by analysing all spectra simultaneously. For the data shown, *k*_ex_ = 206.7 ± 1.4 s^–1^. (c) Correlation of *k*_ex_ in free arginine extracted from D-evolution experiments (*x*-axis) and zz-EXSY experiments (*y*-axis) for temperatures of 258 K, 263 K, 268 K, 273 K, and 278 K.

The evolution of the magnetisations during the D-evolution experiment can be described using the standard Bloch–McConnell equations,^26^ which by integration and Fourier transform give a set of theoretically derived spectra as a function of the exchange rate constant *k*_ex_, the intrinsic transverse relaxation constant *R*_2_, and the difference in chemical shift between the two ^15^N_η_; Δ*ω*. One-dimensional experimental ^15^N slices were extracted from each of the D-evolution planes with different *τ* (Fig. 3a) and fitted to the theoretical spectra in order to obtain the exchange rate for the rotation around the N_ε_–C_ζ_ bond (Fig. 3b and ESI†).

To cross-validate the D-evolution approach, the exchange rate constant, *k*_ex_, was also extracted from two-dimensional ^1^H–^15^N zz-EXSY^27^ experiments at temperatures from 258 K to 278 K, where two separate ^15^N_η_ cross-peaks are observed (Fig. S3, ESI†). Above 278 K separate peaks were not observed for the two ^15^N_η_ nuclei, thus preventing a determination of *k*_ex_ from the zz-EXSY experiment. Nonetheless, the D-evolution experiment is still applicable as long as a change in the ^15^N spectrum is observed for different *τ* delays. A comparison of the exchange rate constants *k*_ex_ obtained from zz-EXSY and from D-evolution experiments shows an excellent agreement (Fig. 3c). It should be noted that it is important to keep the amount of D_2_O as low as possible (<1%) for the extraction of exchange constants using D-evolution-based experiments, since addition of D_2_O artificially increases the obtained *k*_ex_ from D-evolution.

In a second verification of the D-evolution experiment *k*_ex_ rates were obtained from 275 K to 293 K for free arginine in 99%/1% H_2_O/D_2_O at pH 5.5. Here the obtained rates obey a linear Arrhenius relationship (Fig. S4, ESI†) with a transition state enthalpy, Δ*H*^‡^ = 48.7 ± 0.6 kJ mol^–1^, for the N_ε_–C_ζ_ bond rotation of free arginine, which is in good agreement with previous determinations.^28^ The rate for free arginine serves as a point of reference for the rotational dynamics of arginine in an unhindered state. Lower exchange rates for arginine side chains in proteins indicate a hindered rotation around the N_ε_–C_ζ_ bond and hence the presence of interactions formed by the guanidino group.

After having established D-evolution as a method to determine the rate of the ^13^C_ζ_–^15^N_ε_ rotational dynamics we turned our attention back to T4L99A and focussed on residues that are well-resolved in the ^13^C_ζ_–^15^N_η_ spectrum, *i.e.* R14, R52, R95, R96 and R145 (Fig. 2). The remaining arginine side chains show exchange-averaged ^15^N_η_ resonances and are thus expected to experience fast ^13^C_ζ_–^15^N_ε_ rotation and consequently not to form significant interactions. A reference spectrum and D-evolution spectra with six different *τ* delays were recorded at two static magnetic fields (11.7 T and 18.8 T).

Three of the arginine side chains of T4L99A were amenable to analysis with D-evolution, that is, R14, R52 and R96. R145 did not offer sufficient signal-to-noise, while R95 exchanged too slowly for accurate *k*_ex_ to be obtained. For the three arginine side chains amenable to analysis, the exchange rates extracted from the two fields individually were in good agreement (see Fig. S5–S7, ESI†). The precision of the analysis improved when data at both magnetic fields were used, since the correlation between the model parameters decreased. It is noted that the three residues display distinct exchange dynamics. The ^13^C_ζ_–^15^N_ε_ rotation is rapid for R14 (*k*_ex_ = 2000 ± 65 s^–1^ at 298 K, Fig. S5, ESI†) indicating an arginine side chain that does not form interactions, which agrees with the crystal structure of T4L99A (Fig. 4a). This rate is even slightly faster than the rate obtained for free arginine (*k*_ex_ = 1730 s^–1^ at 298 K). The increase in the rate of R14 by roughly 15% corresponds to a decrease in activation energy for the rotation by approximately 0.4 kJ mol^–1^ compared to the free arginine side chain. A characterisation of another surface exposed arginine side chain, R54 in the protein ubiquitin (Table S2, ESI†), shows a similar trend to that observed for R14 in T4L99A, *i.e.*, a slightly faster rate than that observed for free arginine. This could indicate a difference in the protein environment compared to the solvent that results in a slight reduction of the partial double bond character of the N_ε_–C_ζ_ bond or a decreased entropic cost for rearrangement of the solvent shell.

**Fig. 4:**
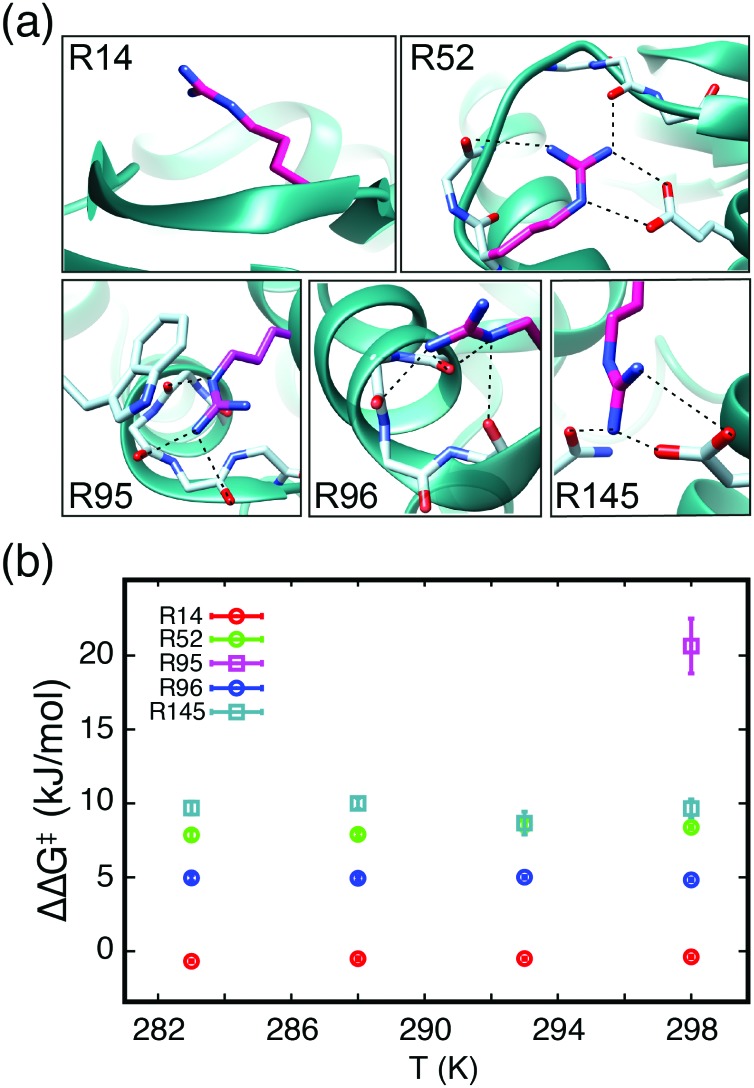
(a) The local environments of R14, R52, R95, R96, and R145 in the crystal structure of T4L99A (pdb: 3dmv). Hydrogen bonds are depicted as dotted lines. (b) ΔΔ*G*^‡^ = Δ*G*^‡^(Arg) – Δ*G*^‡^(free) = –*RT* ln[*k*_ex_(Arg)/*k*_ex_(free)] as a function of temperature for the five arginine side chains in (a). ΔΔ*G*^‡^ for R14, R52 and R96 were obtained using D-evolution (circles), while ΔΔ*G*^‡^ for R95 and R145 were obtained from longitudinal exchange experiments (squares).

The exchange rates for R52 (*k*_ex_ = 57 ± 3 s^–1^ at 298 K, Fig. S6, ESI†) and R96 (*k*_ex_ = 242 ± 8 s^–1^ at 298 K, Fig. S7, ESI†) clearly display a hindered N_ε_–C_ζ_ bond rotation compared to the free arginine and thus show that R52 and R96 are involved in interactions. The crystal structure confirms that both residues are involved in hydrogen bonding networks, see Fig. 4a. The strength of these interactions, which are to be broken upon rotation about the N_ε_–C_ζ_ bond, contribute to an increase of the activation energy for the rotation and explains the reduced exchange rates. The observed rates for N_ε_–C_ζ_ bond rotation follow a linear Arrhenius behaviour, however, the accuracy is not at a level where reliable values of both Δ*H*^‡^ and Δ*S*^‡^ can be obtained and ΔΔ*G*^‡^ relative to the free arginine are therefore reported in Fig. 4b. The ΔΔ*G*^‡^ for R96 of 4.95 ± 0.06 kJ mol^–1^ corresponds approximately to the breakage of one weak hydrogen bond. The ΔΔ*G*^‡^ for R52 of 8.2 ± 0.3 kJ mol^–1^ shows a stronger stabilisation compared to R96 and agrees with the crystal structure, which shows that R52 is forming ionic bidentate hydrogen bonds that are considerably stronger than the hydrogen bonds formed between R96 and the backbone.

A longitudinal exchange method reported previously^24^ using selective inversions was used to probe the rotational dynamics of R95 and R145. For these side chains the two ^15^N_η_ resonances are separated by several ppm and consequently one of the ^15^N_η_ resonances can be inverted selectively. Two longitudinal relaxation experiments were recorded: in the first experiment one of the ^15^N_η_ resonances was inverted, while in the second experiment none of the resonances were inverted. An exchange of the two ^15^N_η_ will lead to an increased longitudinal relaxation in the experiment where one resonance has been inverted.

An analysis of the relaxation decay for R95 gives an exchange rate of 0.4 ± 0.3 s^–1^ (Fig. S8a and b, ESI†) and a stabilisation of 20.7 ± 1.9 kJ mol^–1^ compared to the free arginine. The strong interaction network together with a tryptophan side chain that obstructs the rotation explains this slow rotation around the N_ε_–C_ζ_ bond. For R145 a ΔΔ*G*^‡^ of 9.5 ± 0.5 kJ mol^–1^ was obtained over the temperature range from 283 K to 298 K. This indicates a stabilisation similar to that of R52 and agrees well with the crystal structure, where R145 is also forming ionic bidentate hydrogen bonds. Overall, the obtained ΔΔ*G*^‡^ correlates well with the interactions observed in the crystal structure, which indicates a one-step breakage of the interactions upon rotation around the N_ε_–C_ζ_ bond.

To summarise, we developed an NMR-based approach to quantify the rotational dynamics of the guanidino group of arginine side chains. The method relies on ^13^C-detection and either D-evolution or longitudinal exchange depending on the rate and it is suitable for arginine amine groups whose amine protons exchange rapidly with the solvent. The D-evolution and the longitudinal exchange experiment are complementary and together cover the broad range of exchange rates observed for N_ε_–C_ζ_ bond rotations of arginine side chains. An application to the 19 kDa T4L99A and the agreement of the obtained exchange rates with arginine interaction networks in the crystal structure demonstrates the applicability of this method to monitor interactions such as hydrogen bonding (R52, R95, R96, R145) and cation–π interactions (R95). It is envisaged that the new method serves as a particularly valuable tool to characterise active sites in enzymes, protein–protein or protein–nucleic acid interactions, where arginine residues are expected to play a crucial role.

The Wellcome Trust is acknowledged for supporting the ISMB NMR facility and the Francis Crick Biomedical NMR centre is acknowledged for access to high-field NMR spectrometers. Lucas Siemons is acknowledged for producing the ubiquitin sample. KG acknowledges King's College University and the LiDO programme for a PhD studentship. This research was supported by BBSRC and the Leverhulme Trust.

## Supplementary Material

Supplementary information
