# Supplementary material for: Determining rotational dynamics of the guanidino group of arginine side chains in proteins by carbon-detected NMR
Source: Chem Commun (Camb). 2017 Aug 25;53(72):10062–5. doi: 10.1039/c7cc04821a (PMC5708338; doi:10.1039/c7cc04821a)
Supplement: Supplementary file 1 [file CC-053-C7CC04821A-s001.pdf]

Electronic Supplementary Material (ESI) for ChemComm.
This journal is © The Royal Society of Chemistry 2017

Supporting material for

Determining rotational dynamics of the guanidino group of arginine side chains in proteins by carbon-detected NMR

Karola Gerecht, Angelo Miguel Figueiredo, D. Flemming Hansen

1 Methods

1.1 Protein expression and purification
1.2 NMR Samples
1.3 Longitudinal zz-exchange spectroscopy
1.4 Extraction of the exchange constant k_{ex} from Longitudinal Exchange Spectroscopy
1.5 Divided Evolution (D-Evolution) to characterize exchange processes
1.6 Extraction of k_{ex} from D-Evolution based experiments
1.7 Pulse programme (Bruker)

2 Supplementary Figures and Tables

2.1 Figure S1: Arginine side chain
2.2 Figure S2: Carbon-detected $^{13}C_{\zeta}$-$^{15}N_{\varepsilon}$ HSQC spectrum of T4L99A.
2.3 Figure S3: Extraction of exchange rates for free arginine via longitudinal ^{1}H-^{15}N zz-exchange
2.4 Figure S4: Exchange constants for free arginine as a function of temperature
2.5 Figure S5: Exchange rates for free arginine as a function of temperature
2.6 Figure S6: Extraction of exchange rate for T4L R14 at 298 K using spectra from 500 and 800 MHz together.
2.7 Figure S7: Extraction of exchange rate for T4L R52 at 298 K using spectra from 500 and 800 MHz together.
2.8 Figure S8: Extraction of exchange rate for T4L R96 at 298 K using spectra from 500 and 800 MHz together.
2.9 Figure S9: Longitudinal relaxation of Arg95 for determination of k_{ex}
2.10 Table S1: Rotational exchange rates determined for arginine side chains of T4L99A
2.11 Table S2: Rotational exchange rates and $\Delta\Delta G^{\ddagger}$ determined for R54 of human ubiquitin

1.1 Protein Expression and Purification

Uniformly isotope labelled ^{13}C, ^{15}N T4 lysozyme C54T/C97A/L99A (referred to as T4L99A) was expressed from pET29b(+)-T4L L99A in *E.coli* BL21(DE3) cells. The expression and purification was performed as described previously[1] with minor modifications. Briefly, cultures were grown in 1 L M9 medium (containing 1 g $^{15}NH_4Cl$ and 3 g $^{13}C_6$-glucose as sole nitrogen and carbon source, respectively) at 37 ^oC until they reached an OD_{600} of 0.5 whereafter the temperature was reduced to 18 ^oC. At OD_{600} of 0.7, protein expression was induced with 1 mM IPTG and cultures were grown overnight. Cells were harvested by centrifugation and lysed by sonication. The protein was purified via a 5 mL GE HiTrap SP-FF column, followed by a gel filtration via a 120 mL Superdex 75 column (GE). Fractions containing the protein were combined, buffer exchanged into T4L NMR buffer (50 mM sodium phosphate, 2 mM EDTA, 25 mM NaCl, 2 mM NaN_3, 1 % D_2O, pH 5.5) and concentrated to a final concentration of 1.1 mM (ε_{280}= 25440 M^{-1} cm^{-1}).

Uniformly isotope labelled ^{13}C,^{15}N human ubiquitin was expressed in *E.coli* BL21(DE3) with a cleavable His-tag and purified by standard methods[2,3]. A *ca.* 1.5 mM protein NMR sample prepared in Ubq NMR buffer (20 mM sodium phosphate, 10 mM NaCl, 99%/ 1% H_2O/D_2O, pH 7.4), was used for all studies.

1.2 NMR samples

1) 100 mM arginine in 50 % MeOH, 50 % H_2O; 100% deuterated methanol MeOD-d4 for locking included as an inset.
2) 100 mM arginine in T4L NMR buffer
3) T4 Lysozyme L99A, 1.1 mM in T4L NMR buffer.
4) Human Ubiquitin, ~1.5 mM in Ubq NMR buffer

1.3 Longitudinal Exchange Spectroscopy

Two types of longitudinal exchange experiments were recorded, that is, (1) a ^1H-detected two-dimensional zz-exchange experiment[4] and (2) a ^{13}C-detected one-dimensional exchange experiment using selective pulses[5,6].

The two-dimensional ^1H-detected zz-exchange experiments on free ^{13}C,^{15}N-arginine were recorded on a Bruker Avance III 600 MHz spectrometer equipped with a room temperature TXI probe. The pulse sequence is depicted in Fig S3a. Briefly, an initial INEPT

transfers magnetisation from ^{1}H to ^{15}N resulting in a two-spin order longitudinal $2H_zN_z$ matrix density element. During the following chemical shift evolution, t_1, the ^{15}N chemical shift is encoded. Subsequently, magnetisation is converted to longitudinal magnetisation for the duration of the mixing time τ_m. Finally, magnetisation is transferred back to ^{1}H for detection. Magnetisation that do not exchange during the mixing time give rise to diagonal peaks at {$\omega_1(N_{\eta1})$, $\omega_2(H_{\eta1})$} and {$\omega_1(N_{\eta2})$, $\omega_2(H_{\eta2})$}. Magnetisation that exchange during the mixing time resonate at a different frequency during t_2 detection, and give rise to cross peaks at {$\omega_1(N_{\eta1})$, $\omega_2(H_{\eta2})$} and {$\omega_1(N_{\eta2})$, $\omega_2(H_{\eta1})$}. For each experiment, spectra were recorded for 20 different mixing times τ_m in the range from to 2 to 300 ms. Spectra were analysed using nmrDraw[7] and visualized in CCPNMR[8].

The ^{13}C-detected longitudinal exchange experiments on T4L99A were recorded on a Bruker Avance III HD 800 MHz spectrometer equipped with a cryogenic TCI probe using the pulse sequence in Fig 1c. Two relaxation decay curves were obtained for both Arg95 and Arg145, one with no inversion and one with inversion of either the upfield $^{15}N_\eta$ (Arg95) or the downfield $^{15}N_\eta$ (Arg145) by a 10 ms RE-BURP pulse. Six relaxation delays, T_{relax}, between 0 ms and 512 ms were used for characterising the exchange of Arg95, while eight delays in the range from 0 ms to 300 ms were used to characterise the exchange of Arg145. The sweep width in the ^{15}N dimension was 11 ppm and 12 complex points were recorded. The inter-scan equilibrium delay was 5 s, 160 transients were recorded per FID, which lead to a total acquisition time of 5 hours per plane.

1.4 Extraction of the exchange constant k_{ex} from zz-Exchange Spectroscopy

The ^{1}H-detected 2D longitudinal zz-EXSY experiments were analysed via the intensities of cross and diagonal peaks. These intensities were extracted using FuDA[9] for each of the mixing times by assuming a common line-shape for all mixing times. The time-evolution of longitudinal magentisation was described before[10,11] for a system in symmetrical chemical exchange, *i.e.* identical populations of the two exchanging sites ($p_A = p_B = 0.5$). The ratio between intensities from cross- and diagonal-peaks can be described as a function of the exchange constant k_{ex} with[12,11]

$$A \underset{k_B}{\overset{k_A}{\rightleftarrows}} B \qquad (S1)$$

$$\frac{I_{ab} + I_{ba}}{I_{aa} + I_{bb}} = \frac{1 - \exp(-k_{ex}t)}{1 + \exp(-k_{ex}t)} \qquad \text{(S2)}$$

where k_{ex}=k_A+k_B, I_{ab} and I_{ba} are the intensities of the two cross peaks, and I_{aa} and I_{bb} are the intensities of the diagonal peaks. The exchange rate constant k_{ex} was extracted by using the least squares fitting protocol implemented in gnuplot and fitting the experimental intensities to Eq. S2.

The ^{13}C-detected longitudinal 1D exchange experiments were analysed via the intensities of the two $^{15}N_{\eta}$ resonances as a function of the relaxation delay T_{relax}. In the analysis, the time-evolution of the longitudinal magnetisations were described by:

$$\frac{d}{dt}\begin{pmatrix} I_a \\ I_b \end{pmatrix} = -\mathbf{\Gamma}\begin{pmatrix} I_a \\ I_b \end{pmatrix}, \qquad \text{(S3)}$$

where

$$\mathbf{\Gamma} = \begin{pmatrix} R_1 + k_A & -k_B \\ -k_A & R_1 + k_B \end{pmatrix} \qquad \text{(S4)}$$

Integration of Eq. S4 gives

$$\begin{pmatrix} I_a(t) \\ I_b(t) \end{pmatrix} = \exp(-\mathbf{\Gamma}t)\begin{pmatrix} I_{a,0} \\ I_{b,0} \end{pmatrix} \qquad \text{(S5)}$$

Finally, the best-fit model parameters, $I_{a,0}$, $I_{b,0}$, R_1, and k_{ex} were determined by minimising the target function, χ^2, using in-house written software based on the LMFIT python library[13].

$$\chi^2 = \sum \frac{\left(I_{i,\text{calc}}(t) - I_{i,\text{obs}}(t)\right)^2}{\sigma^2} \qquad \text{(S6)}$$

In eq S6, $i=\{a,b\}$, $I_{i,calc}(t)$ are calculated intensities, $I_{i,exp}(t)$ are the experimentally observed peak intensitites as a function of the relaxation delay T_{relax}, and σ is the uncertainty of the experimentally observed peak intensities.

1.5 Divided Evolution (D-Evolution) experiments to characterise exchange processes

D-Evolution experiments were recorded on Bruker 500 MHz, 600 MHz, and 800 MHz spectrometers using the pulse sequence depicted in Fig 1b. Magentisation was transferred from ^{13}C to ^{15}N via an INEPT sequence with the delay Δ set to $1/(8\,J_{\mathrm{CN}})$ to generate complete antiphase magnetisation. Two pulses ensured selectivity during this process. The shaped E-BURP as the initial $90°_x$ excitation pulse is centred on the $^{13}C_\zeta$ resonance (156 ppm) and the shaped RE-BURP pulse is centred at 70.3 ppm to invert $^{15}N_\eta$, while not inverting $^{15}N_\varepsilon$. After the transfer, each chemical evolution time $1/(2\ \mathrm{SW}) = \delta/2$ is followed by a short CPMG block consisting of two 180° refocusing pulses and of length $T_{\mathrm{CPMG}}=4\tau$ each. For each experiment, 6 to 7 planes of different τ were recorded (typically one reference spectrum without CPMG as well as τ = 68 μs, 125 μs, 250 μs, 500 μs, 750 μs, 1000 μs). Spectra were analysed using the Bruker TopSpin software package, nmrDraw[7], and visualized in CCPNMR[8].

T4 lysozyme: For spectra recorded on T4L99A, a sweep width of 11 ppm was used in the ^{15}N dimension and 32 complex points were recorded. The inter-scan equilibrium delay was 3.3 s. At a temperature of 283K, 216 transients were recorded per FID leading to a total acquisition time of 13 hours per 2D plane. At all other temperatures 152 transients were recorded per FID leading to a total acquisition time of 9 hours per 2D plane.

Ubiquitin: For spectra recorded on Ubiquitin, a sweep width of 9.6 ppm was used in the ^{15}N dimension and 24 complex points were recorded. The inter-scan equilibrium delay was 3.0 s. 8 transients were recorded per FID leading to a total acquisition time of 0.5 hour per 2D plane, except for data recorded at 500 MHz at 278K where 16 transients were recorded per FID. The chemical shifts of the $^{13}C_\zeta$-$^{15}N_\eta$ resonance of R54 were assigned via the $^{13}C_\zeta$ chemical shift observed in a $^{13}C_\zeta$-$^{15}N_\varepsilon$ spectrum. The $^{13}C_\zeta$-$^{15}N_\varepsilon$ spectrum was, in turn, assigned using a ($^{13}C_{\mathrm{aliphatic}}$,$^{13}C_\zeta$) 2D-plane of a CCNeCz-TOCSY experiment[14] and a previous assignment of ubiquitin[15].

1.6 Extraction of k_{ex} from D-Evolution based experiments

The evolution of magnetisation during the D-evolution element is described by the Bloch equations[11,12,16]. Directly after the transfer of magnetisation to $^{15}N_\eta$, the magnetization state can be described by a complex vector, **v** = (0.5, 0.5), since equal transfer to both amine groups is expected, which corresponds to a population of p_A=p_B=0.5. Matlab was used to propagate the magnetisation vector by integration of the Bloch equations. During free precession, the evolution of the transverse component of the magnetisation is described by the following matrix for symmetric exchange:

$$\mathbf{\Gamma} = \begin{pmatrix} R_2 + \frac{k_{ex}}{2} - \frac{1}{2} i\Delta\omega & -\frac{k_{ex}}{2} \\ -\frac{k_{ex}}{2} & R_2 + \frac{k_{ex}}{2} + \frac{1}{2} i\Delta\omega \end{pmatrix} \qquad \text{(S7)}$$

where R_2 is the intrinsic transverse relaxation rate assumed to be identical in the two sites, k_{ex} is the exchange rate constant defined as the sum of the forward and reverse rate constants, and $\Delta\omega$ is the difference in peak position between the two exchanging sites in (rad/sec).

Scalar couplings between $^{15}N_\eta$ and $^{13}C_\zeta$ are refocused during the D-Evolution element due to the 180° ^{13}C pulse and can therefore be ignored. The CPMG 180° pulses refocus the magnetisation, which was considered by taking the complex conjugate of the current magnetization state matrix. The final magnetisation state (the FID) in the ^{15}N dimension was subjected to the same spectra processing as the actual recorded spectra, *i.e.* application of a Lorentzian-to-Gaussian window function followed by a phase correction and zero-filling. Fourier transformation of the calculated FID yielded the theoretically derived spectra as a function of the model parameters described in the Liouvillian $\mathbf{\Gamma}$ in Eq (S7), that is R_2, k_{ex} and $\Delta\omega$. Experimental data were fitted to this set of theoretical spectra via a minimisation of the target function, χ^2,:

$$\chi^2 = \frac{\sum_{i=1}^{N} (expSpec(i) - theorSpec(i))^2}{\sigma^2}$$

with N being the number of points in the dataset. We used the noise estimate of the spectra obtained from nmrDraw for the uncertainty σ. For the experimental data, 1D ^{15}N spectra were extracted for each plane separately (usually three 1D slices were summed up for each peak to obtain higher signal intensity) using FuDA and imported into Matlab. The noise was scaled

according to the number of spectra summed up for each peak. Uncertainties for the obtained model-parameters were calculated using the covariance method[17].

1.7 Pulse programme

```
; Filename: cn_hsqc_arg_dEvol_dfh_800_v2
;
; Written on 14th November 2016 by D Flemming Hansen.
;
; This pulse sequence allows for D-evolution in the indirect 15N
; chemical shift evolution period of arginine 13Cz-15Neta/15Nepsilon
; correlation spectra.
;
; WARNING: This sequence does not work with 'getprosol'. If you by accident
;  type getprosol using this sequence, then all parameters must
;  be checked carefully.
;
; How to:
; =======
;
; INEPT elements with 15N-selective Reburp inversion pulses
;  - For 13Cz-15Neta spectra:
;     . Set the nitrogen carrier at 70.3ppm with a 4.5ms ReBurp inversion
;       pulse at 800 MHz
;     . Set CNST25 [15Nepsilon] to 84.5 ppm
;     . Set effective scalar coupling to CNST2 = 40 Hz, which is 2*JCN.
;       This will generate a density element proportional to
;       2CzNz(eta1) + 2CzNz(eta2) after the first INEPT
;
; D-evolution tauCPMG delays are taken from vdlist. Set F1 TD to the
; number of delays in the vdlist. vd=0 is the reference plane
;  - The high power 15N pulse (pwn,pl3) is used for D-evolution. Therefore,
;    keep pwn>=40us for stability of probe
;
; Selective 13Cz excitation.
;  (pwc_sel:sp12) [1.3 ms EBurp at 800 MHz]
;
; Carbon decoupling with adiabatic inversion or hard pulse in the middle
;  of the nitrogen chemical shift evolution.
;  (pwc_chirp:sp13) [500us Chirp]
;
; 1H decoupling during indirect evolution & acquisition
;  [65us WALTZ64 decoupling]
;

;$CLASS=HighRes
;$DIM=3D
;$TYPE=
;$SUBTYPE=
;$COMMENT=

#include <Avance.incl>
#include <Delay.incl>
#include <Grad.incl>

;
; Define pulse lengths
define pulse pwc
  "pwc=p1"
```

```
define pulse pwc_sel
   "pwc_sel=p12"

define pulse pwc_chirp
   "pwc_chirp=p13"

define pulse pwn
   "pwn=p3"

define pulse pwn_sel
   "pwn_sel=p31"
;
; Define delays
define delay taua
   "taua=1s/(cnst2*4)"

define delay eta
define delay tt

"d11=30m"
"d12=2u"
"d13=2u"

"in0=inf2/2"
"tt=in0"

"cnst21=o1/bf1"
"cnst23=0.5*(cnst21+cnst22)"
"cnst24=o3/bf3"
"cnst26=0.5*(cnst24+cnst25)"

#ifdef HALFDWELL
   "d0=in0/2-0.5*pwc_chirp-0.63662*pwn"
#else
   "d0=in0-0.5*ppwc_chirp-0.63662*pwn"
#endif /*HALFDWELL*/

"spoal12=1.0"
"spoffs12=0.0"

"spoal13=0.5"
"spoffs13=0.0"

"spoal31=0.5"
"spoffs31=0.0"

;
; Automatic calculation of powers for selective pulses. Not that the
; powers have been optimized for best performance of Eburp and Reburp
; pulses, which is not a perfect inversion at zero frequency, and not
; using the integration factors in the headers.
#ifdef AUTOCAL
   "spw12=(pwc/pwc_sel)*(pwc/pwc_sel)*plw1/(0.05813*0.05813)"
   "spw31=(pwn/pwn_sel)*(pwn/pwn_sel)*plw3/(0.041920*0.041920)"
   "plw32=(pwn/pcpd3)*(pwn/pcpd3)*plw3"
#endif /* AUTOCAL */

;
; Hard-code zero powers
"plw0=0"
"plw30=0"

;
```

```
;loop the tauCPMG (vd-list) first
aqseq 312

1 ze
;
; Do some safety checking
;
  if "td2>64"
  {
    2u
    print "To many points in the indirect dimension"
    goto HaltAcqu
  }
  if "vd < 68u && vd > 0.1u"
  {
    2u
    print "tauCPMG is too short" goto HaltAcqu
  }
  if "pwn<39u"
  {
    2u
    print "15N pulse too short" goto HaltAcqu
  }
  if "pcpd3<300"
  {
    2u
    print "15N decoupling power too high" goto HaltAcqu
  }

  d12
2 d11 do:f2 do:f3

  d12 d1
  d12 pl0:f1 pl21:f2 pl3:f3
  30u fq=cnst21(bf ppm):f1
  30u fq=cnst24(bf ppm):f3

  50u UNBLKGRAD
;
; 15N purge pulse
  (pwn ph10):f3

  2u
  p51:gp1
  d16

;
; The real start of the pulse sequence
  (pwc_sel:sp12 ph10):f1
  d12 pl1:f1 pl30:f3

  2u
  p52:gp2
  d16

  "DELTA=taua-d12-2u-p52-d16-larger(pwc,pwn_sel*0.5)"
  DELTA

  ( center (pwc*2. ph10):f1 (pwn_sel:sp31 ph10):f3 )

  2u
  p52:gp2
  d16
```

```
  d12 pl3:f3

  "DELTA=taua-2u-p52-d16-d12-0.6366*pwc-larger(pwc,pwn_sel*0.5)"
  DELTA

  (pwc ph1):f1

  2u
  p53:gp3
  d16

  d12 pl0:f1 pl21:f2 pl3:f3
  30u fq=cnst23(bf ppm):f1

;
; start proton decoupling and reset the phase for XY16 CPMG pulse (ph25)
  d13 cpd2:f2 ph10 rpp25

  "COUNTER=trunc(d0/tt+0.5)"
  "eta=d0-COUNTER*tt"

  (pwn ph4):f3
  eta
  if "COUNTER>0"
  {
6   tt
    if "vd>0"
    {
      "DELTA=vd-pwn"
      DELTA
      (pwn*2. ph25):f3
      DELTA ipp25
      DELTA
      (pwn*2. ph25):f3
      DELTA  ipp25
    }
    lo to 6 times COUNTER
  }

  (pwc_chirp:sp13 ph10):f1
  if "COUNTER>0"
  {
7   tt
    if "vd>0"
    {
      "DELTA=vd-pwn"
      DELTA
      (pwn*2. ph25):f3
      DELTA ipp25
      DELTA
      (pwn*2. ph25):f3
      DELTA ipp25
    }
    lo to 7 times COUNTER
  }
  eta
  (pwn ph5):f3

  d13
  d13 do:f2
  30u fq=cnst21(bf ppm):f1
```

```
  d12 pl1:f1 pl30:f3

  2u
  p54:gp4
  d16

  (pwc ph10):f1

  2u
  p55:gp5
  d16

  "DELTA=taua-pwc*0.6366-2u-p55-d16-larger(pwc,pwn_sel*0.5)"
  DELTA

  ( center (pwc*2. ph10):f1 (pwn_sel:sp31 ph10):f3 )

  2u
  p55:gp5
  d16

  "DELTA=taua-2u-p55-d16-larger(pwc,pwn_sel*0.5)-30u-d12-d13"
  DELTA

;
; Move 15N carrier to middle of 15Neta,15Nepsilon range
  30u fq=cnst26(bf ppm):f3

;
; Set powers for decoupling
  d12 pl21:f2 pl32:f3
  d13 BLKGRAD

  go=2 ph31 cpd2:f2 cpd3:f3
  d11 do:f2 do:f3 mc #0 to 2
    F1QF(ivd)
    F2PH(ip4, id0)

HaltAcqu, 1m exit

ph1= 1 1 1 1  3 3 3 3
ph4= 0 2
ph5= 0 0 2 2

ph10=0
ph11=1
ph12=2
ph13=3

ph25=0 1 0 1  1 0 1 0  2 3 2 3  3 2 3 2

ph31= 0 2 2 0  2 0 0 2

;pwc : 13C 90 degree hard pulse width
;pwc_sel: 13C selective excitation
;pwc_chirp: 13C inversion during 15N chemical shift evolution
;pwn : 15N 90 degree hard pulse width
;pwn_sel :      15N selective inversion
;pl1 : f1 channel - high power with pwc
;pl2 : f2 channel - high power with pwh
;pl3 : f3 channel - high power with pwn
;pl32: f3 channel - decoupling power level
;p1  : pwc - f1 channel 90 degree pulse width
```

```
;p12 : pwc_sel      [1300us]
;p13 : pwc_chirp [500us]
;p3  : pwn - f3 channel 90 degree pulse width
;p31 : pwn_sel      [4500us]
;p51 : Length of gradient 1 [1000us]
;p52 : Length of gradient 2 [1000us]
;p53 : Length of gradient 3 [1000us]
;p54 : Length of gradient 4 [1000us]
;p55 : Length of gradient 5 [1000us]
;spnam12: 13C selective excitation [Eburp2.1000]
;spnam13: 13C inversion                   [Crp80,0.5,20,1]
;spnam31: 15N selective inversion         [Reburp.1000]
;cnst2: = J(NC)      [20 Hz for Nepsilon, 40Hz for Neta]
;cnst21: C_zeta chemical shift offset [= o1p, 156 ppm]
;cnst22: C_delta chemical shift offset [40.5 ppm]
;cnst24: N_eta chemical shift offset [= o3p,70.3 ppm]
;cnst25: N_epsilon chemical shift offset [84.5 ppm]
;d16: Gradient recovery delay [200us]
;o1p: C_zeta chemical shift offset [156 ppm]
;o2p: H_epsilon/H_eta chemical shift offset [7 ppm]
;o3p: N_eta chemical shift offset [70.3 ppm]
;inf1: 1/SW(X) = 2 * DW(X)
;in0:  1/(2 * SW(X)) = DW(X)
;NS: 8 * n
;DS: 64
;td1: number of delays in vd-list
;td2: number of experiments in F2
;FnMODE: QF in F1
;FnMODE: States-TPPI, TPPI, States or QSEQ in F2
;cpd2: decoupling according to sequence defined by cpdprg2: WALTZ64
;pcpd2: f2 channel - 90 degree pulse for decoupling sequence
;cpd3: decoupling according to sequence defined by cpdprg3: garp4

;for z-only gradients:
;gpz1: 17 %
;gpz2: 7 %
;gpz3: 47 %
;gpz4: 33 %
;gpz5: 13 %

;Gradient files:
;gpnam1: SINE.100
;gpnam2: SINE.100
;gpnam3: SINE.100
;gpnam4: SINE.100
;gpnam5: SINE.100
;gpnam6: SINE.100

;preprocessor-flags-start
;HALFDWELL: for initial sampling delay of half a dwell-time with
;              option -DHALFDWELL (eda: ZGOPTNS)
;AUTOCAL: for automatic calculation of powers for shaped pulses
; this assumes perfect linearity of the amplifiers
;              option -DAUTOCAL
;preprocessor-flags-end
```

2 Supplementary Figures

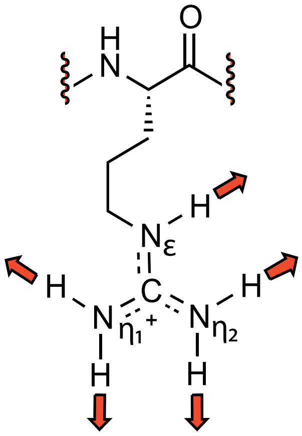

Figure S1: The arginine side chain with red arrows indicating possible side-chain sites for hydrogen bonding interactions.

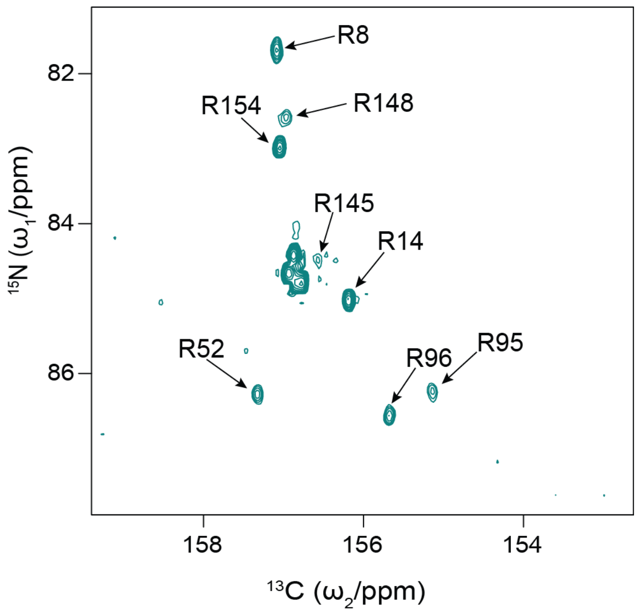

Figure S2: Carbon-detected $^{13}C_{\zeta}$-$^{15}N_{\varepsilon}$ HSQC spectrum of T4L99A. The spectrum was recorded at a field of 18.8 T at 298 K. The assignment shown is taken from ref[14] and it was used to assign the $^{13}C_{\zeta}$-$^{15}N_{\eta}$ spectrum in Fig 1 via the $^{13}C_{\zeta}$ chemical shifts.

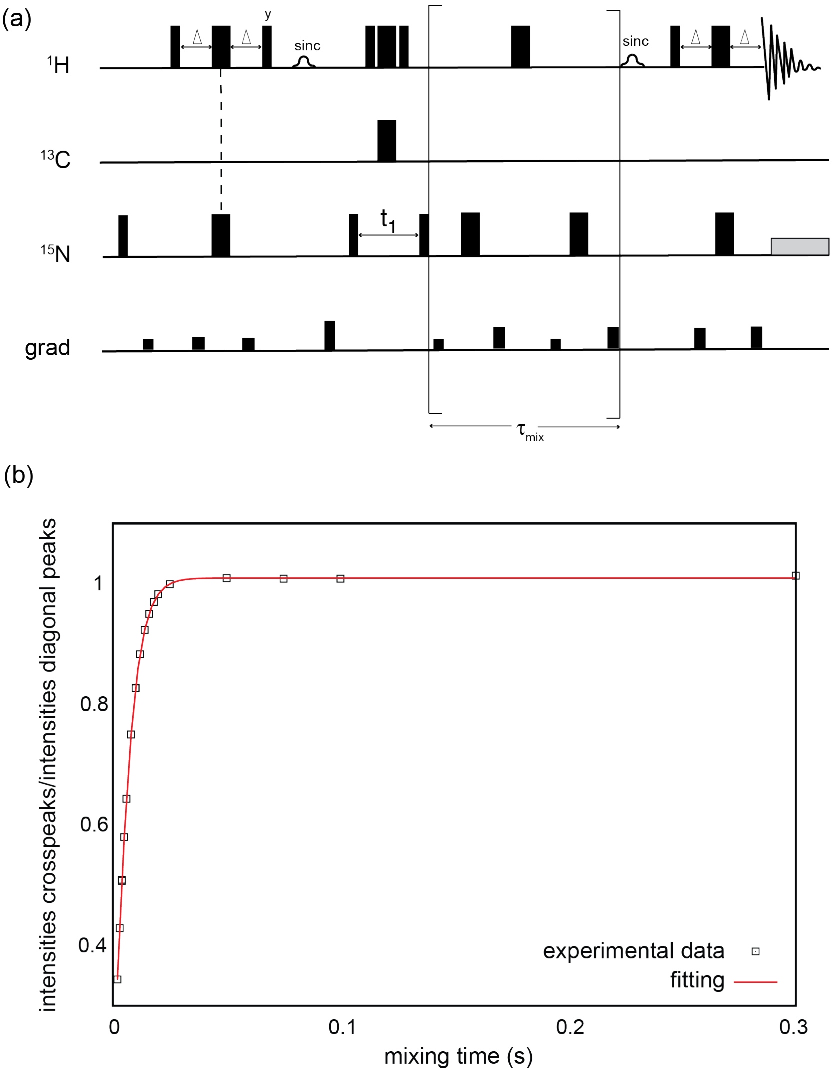

Figure S3: (a) Schematic representation of the longitudinal ^{1}H-^{15}N zz-exchange experiment used to obtain rotational dynamics of free arginine. The carrier positions are: ^{1}H 7 ppm; ^{13}C: 156 ppm, ^{15}N 71 ppm. Narrow bars represent 90° pulses and wide bars represent 180° pulses and the 'sinc' pulse is a shaped water-selective pulse. The delay $\Delta = 1/(4J_{HN}) = 2.8$ ms. ^{15}N decoupling during acquisition was achieved using a WALTZ16 scheme applied at a field of 0.7 kHz. (b) Extraction of the exchange rate for free arginine (100 mM in 50 % H2O/50% MeOH) using longitudinal ^{1}H-^{15}N zz-exchange spectra recorded at 600 MHz at 273 K. Intensities of crosspeaks and diagonal peaks were extracted using FuDA[9]. The ratios of crosspeak intensity to diagonal peak intensity as a function of the mixing time τ_{mix} are plotted in black. An exchange rate k_{ex} of 200.1 ± 0.6 s^{-1} was obtained by least-square fitting (red).

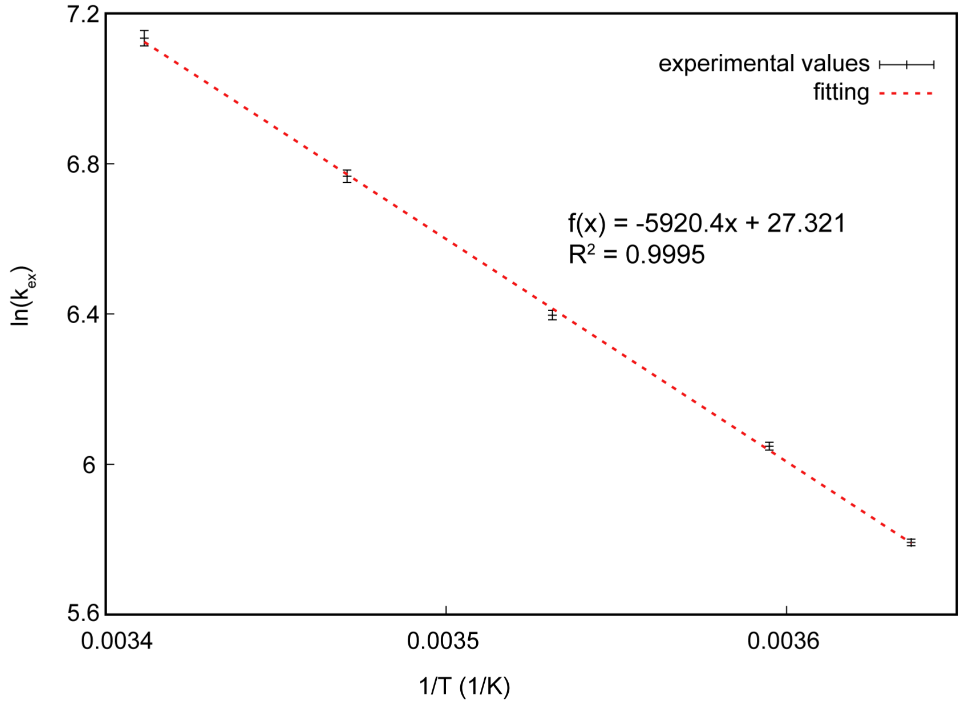

Figure S4: Exchange constants for free arginine in the temperature range from 275 to 293 K, measured using D-evolution. Spectra were recorded at 500 MHz and analysed as described above to extract the exchange rates. ln(k_{ex}) is plotted *versus* 1/T and shows a linear relationship as expected based on the Arrhenius equation. From the linear fit, $\Delta H^{\ddagger}$ = 48.7±0.6 kJ/mol (11.64 ± 0.15 kcal/mol)

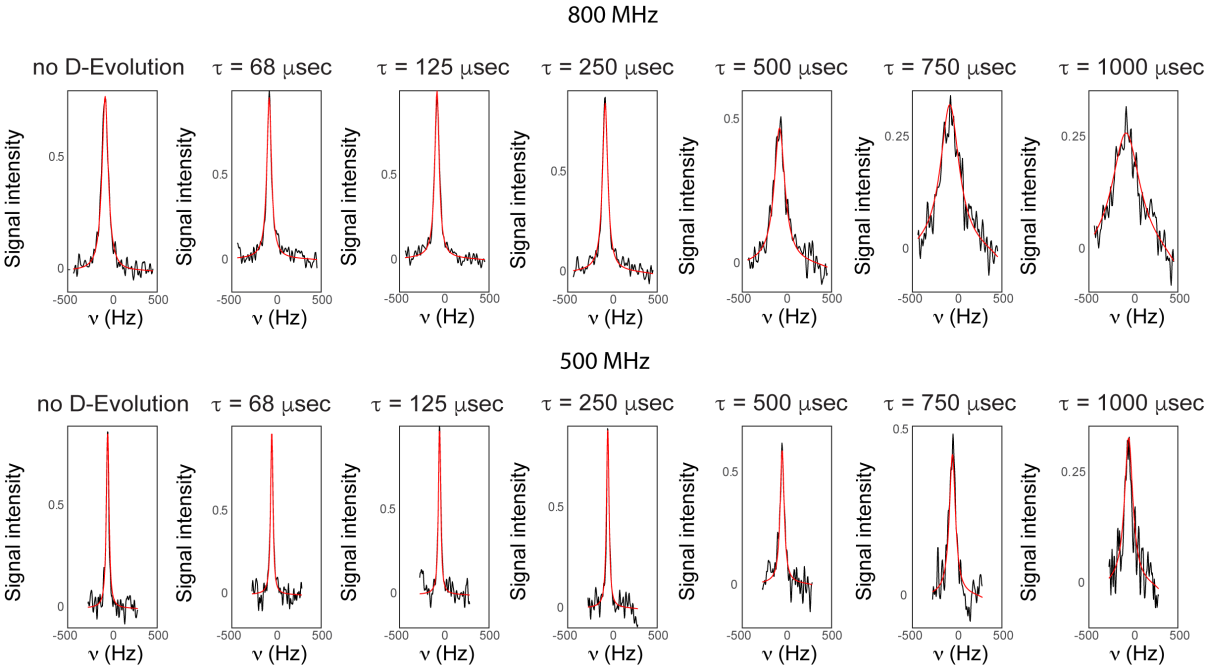

Figure S5: Extraction of exchange rate for T4L R14 at 298 K by a simultaneous analysis of spectra from 500 and 800 MHz. Black lines correspond to the experimental 1D spectra extracted along the $^{13}C_\zeta$ chemical shift of R14 and red lines are results of the least-squares fit.

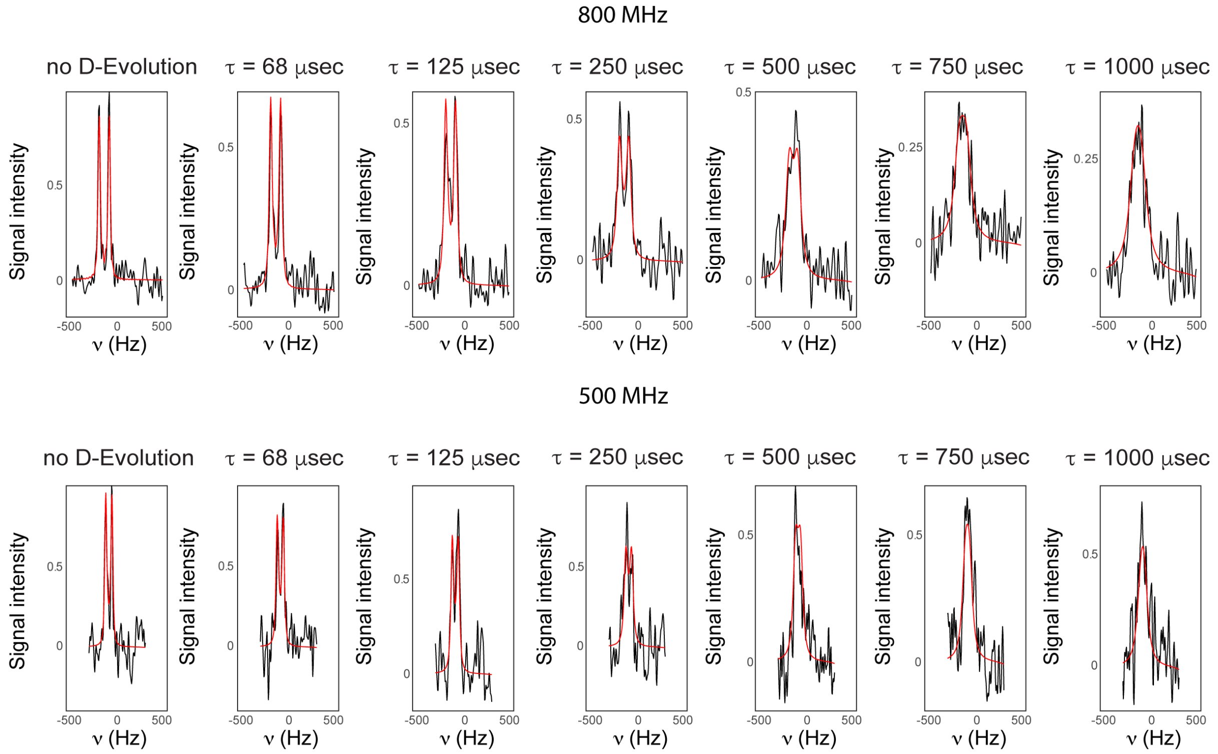

Figure S6: Extraction of exchange rate for T4L R52 at 298 K by a simultaneous analysis of spectra from 500 and 800 MHz. Black lines correspond to the experimental 1D spectra extracted along the $^{13}C_{\zeta}$ chemical shift of R52 and red lines are results of the least-squares fit.

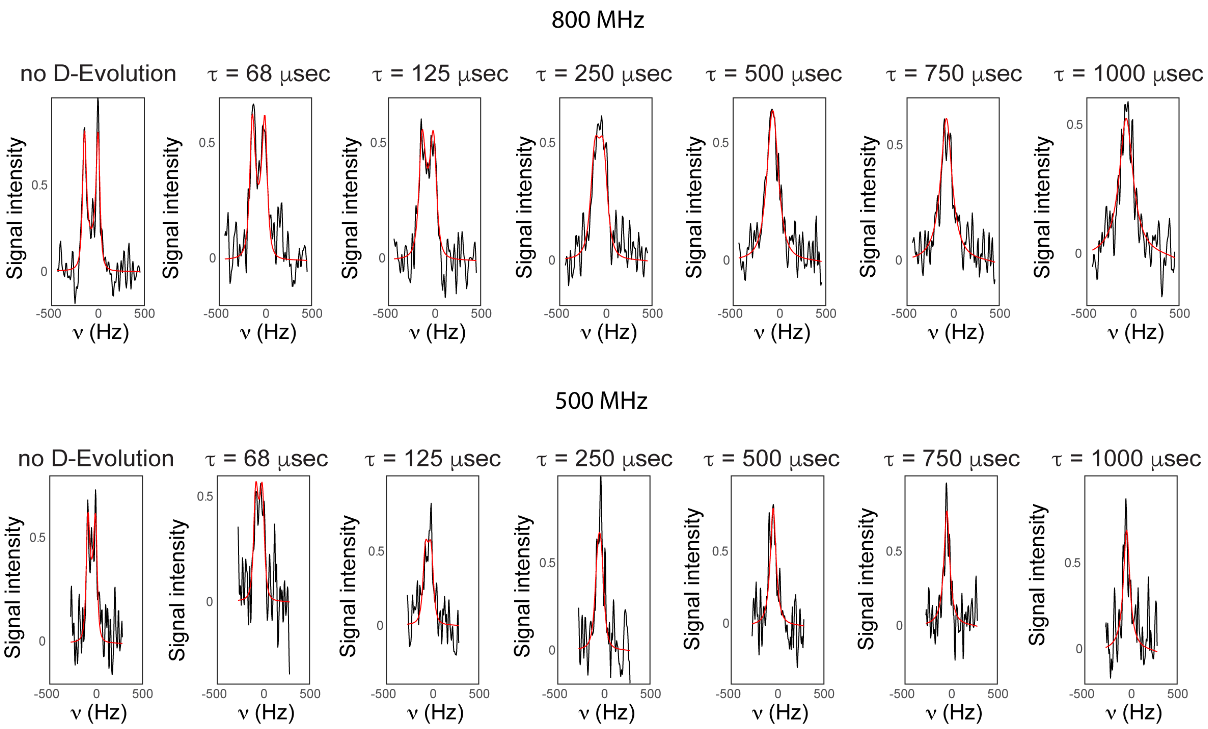

Figure S7: Extraction of exchange rate for T4L R96 at 298 K by a simultaneous analysis of spectra from 500 and 800 MHz. Black lines correspond to the experimental 1D spectra extracted along the $^{13}C_{\zeta}$ chemical shift of R96 and red lines are results of the least-squares fit.

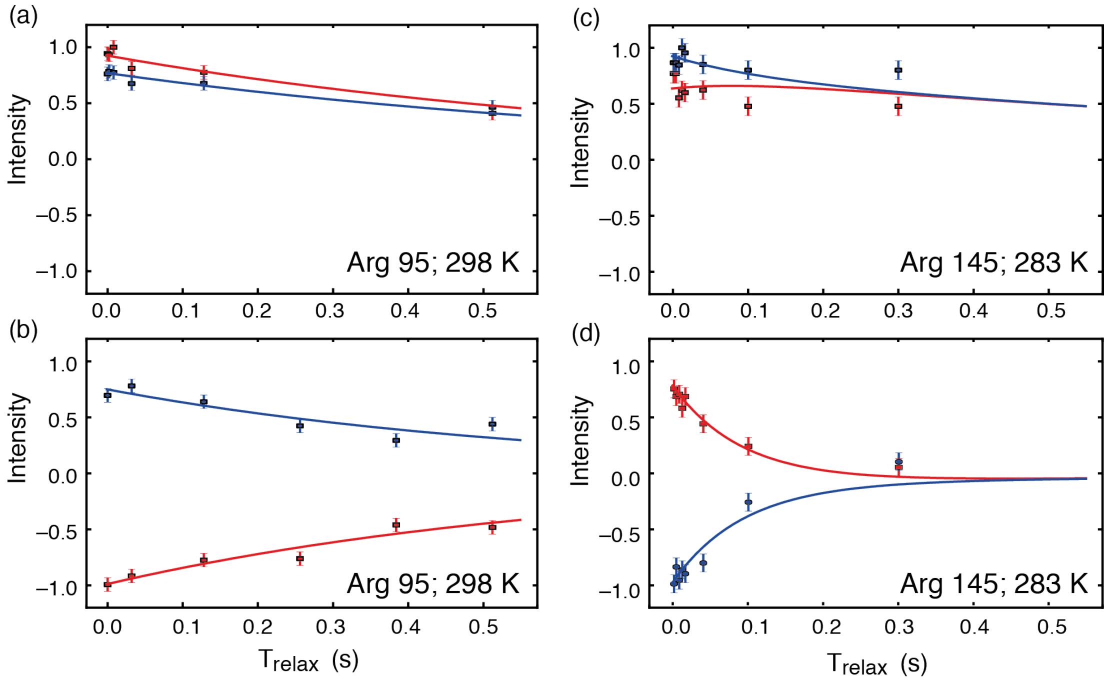

Figure S8: Longitudinal relaxation of Arg95 and Arg145 for determination of k_{ex}. Red squares represent normalised intensities for the upfield $^{15}N_{\eta}$, blue circles represent intensities of the downfield $^{15}N_{\eta}$ resonance, while lines are the result of the least-squares fit. The data shown were recorded at a static magnetic field strength of 18.8 T. (a) Relaxation decay without inversion of the upfield $^{15}N_{\eta}$ resonance of Arg95; (b) Relaxation decay with inversion of the upfield $^{15}N_{\eta}$ resonance of Arg95. The result of the least squares fit for Arg95 (a) and (b) is: k_{ex} = 0.36 ±0.26 s^{-1} and R_1= 1.26 ± 0.20 s^{-1}. (c) Relaxation decay without inversion of the downfield $^{15}N_{\eta}$ resonance of Arg145; (d) Relaxation decay with inversion of the downfield $^{15}N_{\eta}$ resonance of Arg95. The result of the least squares fit for Arg145 (c) and (d) is: k_{ex} = 9.7 ±1.8 s^{-1} and R_1= 1.10 ± 0.52 s^{-1}.

Table S1: Rotational exchange rates determined for arginine side chains of T4L99A

Temp (K)	R14[a)] k_{ex} (s^{-1})	R52[a)] k_{ex} (s^{-1})	R95[b)] k_{ex} (s^{-1})	R96[a)] k_{ex} (s^{-1})	R145[b)] k_{ex} (s^{-1})
**283**	803±23	21.0±2.8	-	72.6±6.6	9.7±1.8
**288**	1069±48	31.5±2.4	-	109.1±6.9	13.1±2.0
**293.2**	1502±46	36.2±2.0	-	155.7±5.2	34.4±11.0
**298**	2000±65	57.3±2.5	0.4 ± 0.3	242.4±8.3	34.3±8.5

a) From simultaneous analysis of D-evolution experiments at 11.7 T and 18.8 T.

b) From longitudinal exchange experiments recorded at 18.8 T.

Table S2: Rotational exchange rates and $\Delta\Delta G^{\ddagger}$ determined for R54 of human ubiquitin

Temp (K)	R54[a)] k_{ex} (s^{-1})	R54[b)] $\Delta\Delta G^{\ddagger}$ (kJ/mol)
**278**	565±35	−0.72±0.14
**288**	1134±53	−0.65±0.11
**298**	1852±90	−0.20±0.12

a) From simultaneous analysis of D-evolution experiments at 11.7 T and 18.8 T.

b) Calculated as described in Fig. 4.

References:

1 P. Vallurupalli, D. F. Hansen, P. Lundström and L. E. Kay, *J. Biomol. NMR*, 2009, **45**, 45–55.

2 D. L. Distefano and A. J. Wand, *Biochemistry*, 1987, **26**, 7272–7281.

3 D. F. Hansen, D. Yang, H. Feng, Z. Zhou, S. Wiesner, Y. Bai and L. E. Kay, *J. Am. Chem. Soc.*, 2007, **129**, 11468–11479.

4 N. A. Farrow, O. Zhang, J. D. Forman-Kay and L. E. Kay, *J. Biomol. NMR*, 1994, **4**, 727–34.

5 J. J. Led and H. Gesmar, *J. Magn. Reson.*, 1982, **49**, 444–463.

6 L. E. Kay, D. A. Torchia and A. Bax, *Biochemistry*, 1989, **28**, 8972–8979.

7 F. Delaglio, S. Grzesiek, G. W. Vuister, G. Zhu, J. Pfeifer and A. Bax, *J. Biomol. Nmr*, 1995, **6**, 277–293.

8 W. F. Vranken, W. Boucher, T. J. Stevens, R. H. Fogh, A. Pajon, M. Llinas, E. L. Ulrich, J. L. Markley, J. Ionides and E. D. Laue, *Proteins Struct. Funct. Bioinforma.*, 2005, **59**, 687–696.

9 D. F. Hansen, D. Yang, H. Feng, Z. Zhou, S. Wiesner, Y. Bai and L. E. Kay, *J Am Chem Soc*, 2007, **129**, 11468–11479.

10 A. G. Palmer, C. D. Kroenke and J. P. Loria, *Meth Enzym.*, 2001, **339**, 204–238.

11 D. F. Hansen and J. J. Led, *J Magn Reson*, 2003, **163**, 215–227.

12 H. M. McConnell, *J. Chem. Phys.*, 1958, **28**, 430–431.

13 M. Newville, T. Stensitzki, D. B. Allan, A. Ingargiola, J. J. Helmus, E. O. Le Bigot, A. R. Nelson and J, 2014: https://lmfit.github.io/lmfit-py/

14 N. D. Werbeck, J. Kirkpatrick and D. F. Hansen, *Angew. Chem. Int. Ed. Engl.*, 2013, **52**, 3145–7.

15 G. A. Lazar, T. M. Handel, E. C. Johnson and J. R. Desjarlais, *Protein Sci.*, 2008, **8**, 2598–2610.

16 G. Bouvignies, D. Hansen, P. Vallurupalli and L. Kay, *J. Am. Chem. Soc.*, 2011, **133**, 1935–1945.

17 W. H. Press, S. A. Teukolsky, W. T. Vetterling and B. P. Flannery, *Numerical Recipes in C*, 1992, vol. 29.
